# Characteristics and risk factors for symptomatic *Giardia lamblia *infections in Germany

**DOI:** 10.1186/1471-2458-10-41

**Published:** 2010-01-28

**Authors:** Werner Espelage, Matthias an der Heiden, Klaus Stark, Katharina Alpers

**Affiliations:** 1 Department for Infectious Disease Epidemiology, Robert Koch Institute, Berlin, Germany

## Abstract

**Background:**

In developed countries, giardiasis is considered a travel related disease. However, routine surveillance data from Germany indicate that >50% of infections were acquired indigenously. We studied the epidemiological characteristics of symptomatic *Giardia *infections acquired in Germany and abroad, and verified the proportion of cases acquired in Germany in order to investigate risk factors for sporadic autochthonous *Giardia *infections.

**Methods:**

We identified *Giardia *cases notified by 41 local health authorities between February 2007 and January 2008 and interviewed them on their clinical symptoms, underlying morbidities, travel abroad and potential risk factors for the disease. We conducted a case-control-study including laboratory-confirmed (microscopy or antigen-test) autochthonous *Giardia *cases with clinical manifestations (diarrhoea, cramps, bloating) and randomly selected controls from the local population registry matched by county of residence and age-group (0-5, 6-19, ≥20 years). Secondary cases, controls with diarrhoea and persons who had travelled outside Germany in the three weeks prior to disease onset (exposure period) were excluded. We calculated adjusted odds ratios (aOR) with 95% confidence intervals (CI) using conditional logistic regression.

**Results:**

Of 273 interviewed cases, 131 (48%) had not travelled abroad during the defined exposure period. Of these 131, 85 (65%) were male, 68 (54%) were living in communities with >100,000 inhabitants and 107 (83%) were aged 20 years or older. We included 120 cases and 240 controls in the case-control study. Cases were more likely to be male (aOR 2.5 CI 1.4-4.4), immunocompromised (aOR 15.3 CI 1.8-127) and daily consumers of green salad (aOR 2.9 CI 1.2-7.2). Contact with animals (pets/farm animals) and exposure to surface water (swimming/water sports) were not associated with symptomatic disease.

**Conclusions:**

A substantial proportion of *Giardia lamblia *cases in Germany are indigenously acquired. Symptomatic cases are significantly more likely to be immunocompromised than control persons from the general population. Physicians should consider *Giardia *infections among patients with no recent history of travel abroad, particularly if they have immune deficiencies. Green salads may be an important vehicle of infection. Information campaigns highlighting this food-borne risk should emphasise the risk to persons with immune deficiencies.

## Background

*Giardia lamblia *is an enteric protozoan pathogen found in a variety of mammalian hosts, including humans. *Giardia lamblia *isolates exhibit wide genetic diversity. Strains isolated from humans can be grouped into the major assemblages A and B [1]. Attempts to associate symptoms with genotype in human infections have not provided a definitive answer to date [2-4].

In humans, *Giardia lamblia *infections have a wide clinical spectrum ranging from asymptomatic carriage to long-lasting diarrhoea with malabsorption. So far, it remains unclear whether asymptomatic human infections relate to the carriage of "non-pathogenic" strains, or whether the host is able to maintain parasite numbers at a subclinical level without complete clearance of the infection. For symptomatic disease the incubation period is usually 3-20 days, but can be much longer. Among healthy immunocompetent individuals the infection is frequently self-limited [5]. Prolonged infections with a high level of post-infectious fatigue and symptoms indistinguishable from those of irritable bowel syndrome have been reported [6].

*Giardia lamblia *is considered to be the most common human protozoan enteropathogen worldwide [7]. In Germany, acute *Giardia lamblia *infections were made notifiable in 2001. Laboratories notify the diagnosis to the local health authorities, who obtain additional information from the cases on age, sex, symptoms, occupation as a food handler and probable place of infection (based on travel abroad during the exposure period). In 2007, a total of 3651 cases of giardiasis were notified to the Robert Koch Institute. Most cases (62%) were reported to have acquired their infection in Germany [8]. Among these cases, the source of infection and possible risk factors were unknown.

It is well documented that in developing countries, infections are associated with poor sanitary conditions, poor water quality and overcrowding [9], whereas in industrialized countries cases are usually associated with international travel and immigration [10]. Populations at increased risk of autochthonous infection include small children in day care centres [11-13], men who have sex with men [14-18] and persons in custodial institutions [19,20].

Large community outbreaks have been attributed to contaminated drinking water [21-24], whereas in smaller outbreaks contaminated food [25-29] and contact with contaminated recreational waters, including swimming and wading pools [30-33], have been implicated.

Little is known about the epidemiology and risk-factors for sporadic human giardiasis in developed countries. A case-control study in the UK reported that swallowing water while swimming, recreational contact with fresh water, drinking tap water and eating green salad were associated with illness [29].

We aimed to describe the epidemiological characteristics of symptomatic *Giardia *infections in Germany, to verify the proportion of cases acquired in Germany and to identify possible risk factors for sporadic symptomatic infections in order to target intervention measures.

## Methods

### Enhanced Surveillance

In 2007, we recruited 41 out of 450 local public health authorities in Germany as sentinel sites for enhanced *Giardia *surveillance. Sentinel public health authorities were selected on the basis of the total number of health authorities per federal state, the population in each federal state, the incidence of notified diseases, the demographic characteristics of the catchment area (rural, urban, both) and number of staff employed by the authority [34] We applied the case definition that is in place for routine *Giardia *surveillance in Germany [35]: (1) laboratory confirmation by microscopy or antigen-test or (2) an epidemiological link to a laboratory-confirmed case with at least one of the following: diarrhoea, abdominal cramps or bloating.

We asked staff at the sentinel health authorities to contact all cases notified to them between February 1st, 2007 and January 31st, 2008. Cases who gave their verbal informed consent were interviewed using a standardised questionnaire, which collected information on diagnosis (date of onset, symptoms, laboratory tests performed and reason for being tested), disease severity (weight loss, days missing at work or in school, hospitalisation), migration status, residence (population at residence, urban or rural residence, living on a farm, owning animals), profession, and host factors related to susceptibility (use of antacids and impaired immunity, e.g. HIV, cancer or organ transplant recipient).

We defined the exposure period as three weeks before onset of symptoms. For this period we asked questions on travel abroad, contact with persons (number and age of household members, caring for small children, elderly or handicapped persons), food history (green salad, raw vegetables, water), contact with surface water (swimming, water sports) and contact with animals (pets, farm animals). In order to verify whether cases might have acquired their infection prior to the defined exposure period, an additional question on foreign travel during the 12 months preceding the defined exposure period of three weeks was included.

Travel destinations were classified according to geographical regions and defined as high-risk (Central America, South America, Asia, Northern Africa and sub-Saharan Africa) or low-risk (Europe, Northern America, Australia and New Zealand) for contracting *Giardia*.

We described all cases with known onset of symptoms and information on travel in the exposure period and compared cases who had travelled abroad during the exposure period ("travel-associated cases") to cases without travel history during that period ("autochthonous cases").

### The case-control study

We conducted a case-control study among autochthonous, lab-confirmed *Giardia *cases with two matched controls per case. Cases who had contact with a laboratory-confirmed notified *Giardia *case during their exposure time (secondary cases) were excluded. Controls were matched by county of residence and age group (0-5, 6-19 and ≥20 years). We selected controls randomly from the cases' local population registry offices. If residents had a publicly listed telephone number attempts were made to contact them on at least three occasions, during the day, evenings and weekends. The interview was conducted either immediately or later at a more convenient time. Controls were excluded if they had diarrhoea or foreign travel during the exposure period. We collected data on the controls' exposure to risk factors for infection during the three weeks prior to the onset of disease in the matched case. Some questions were related to the 12 months preceding that period.

### Statistical analysis

Data were entered into Adobe Acrobat forms (Adobe Systems Incorporated), extracted as xml-files and exported to STATA (Intercooled Stata 10 for Windows, StataCorp LP, College Station, TX, U.S.A.). We compared the characteristics of cases with autochthonous and travel-associated *Giardia *infection using Fisher's exact test for categorical and the Wilcoxon rank sum test for continuous variables. A p-value of less than 5% was considered to be statistically significant.

We selected a sample size of 120, since at least 107 cases are needed to detect an odds ratio of 2.5 for the variable under investigation with 95% confidence and a power of 90%, assuming 10% exposure in the control group and a case-control ratio of 1:2. Using univariate matched analysis, we compared the prevalence of specific exposures between cases and controls, and calculated the corresponding Mantel-Haenszel odds ratios and 95% confidence intervals (CI). Age strata (0-5, 6-19 and ≥20 years) were analysed separately.

We conducted a multivariate analysis using conditional logistic regression models. We defined exposures with a p-value of ≤0.2 in univariate analysis as significant for inclusion in the first step of the model. A stepwise backward removal approach was employed for the sequential removal of insignificant variables. Cases or controls with missing values in one of the variables under analysis were excluded from the model. We applied the likelihood-ratio test to identify the model of best fit, based on maximum likelihood. Different interaction terms were included to test for effect-measure modification.

This epidemiological study was performed in compliance with the Helsinki Declaration. It was conducted under the provision of the German Protection against Infections Act (IfSG). The study was performed in accordance with the standards for data protection established at the Robert Koch Institute, Germany.

## Results

### Enhanced Surveillance

Of 597 *Giardia *infections notified to the routine surveillance system of the sentinel local health authorities between 01 February 2007 and 31 January 2008, 505 were laboratory-confirmed cases; six of these had an epidemiological link to another case. Moreover, 86 cases were excluded since they either did not meet the clinical case definitions [49] or their clinical presentation was unknown [37]. Of all laboratory-confirmed cases, 326 (65%) consented to participate in the study and were interviewed by the local health authority. In 273 (84%) of these cases the specific date of onset of symptoms was known, enabling the exposure period to be defined.

Among 131 autochthonous cases, 15 (11%) reported having had contact with another person with diarrhoea during the exposure period; however, none of these cases was aware of their own diagnosis being secondary to contact with a laboratory-confirmed *Giardia *case.

In our enhanced surveillance the median age of cases with information on travel in the exposure period was 38 years (range 0-92); 153/273 cases (56%) were male. This corresponded to 2150/3768 (57%) males and a median age of 38 years (range 0-93) among those captured in the routine surveillance in Germany during the corresponding period (source: SurvNet@RKI).

### Characteristics of travel-associated vs. autochthonous *Giardia *cases

Of the cases with information on travel during the exposure period, 142 (52%) had travelled abroad (Table 1). Travel-associated cases were more likely to have travelled to high-risk destinations in the 12 months prior to the defined incubation period than autochthonous cases (p < 0.001). The median age of travel-associated cases was 35 years; 48% were male. The median age of autochthonous *Giardia *cases was 41 years (p = 0.046); 65% were male (p = 0.007). Considering that the usual age when people start to work is above 16 years and working ends by the age of 65, the employment status did not differ significantly between travel-associated and autochthonous cases (78% versus 73%; p = 0.394).

**Table 1:** Demographic characteristics and clinical manifestations of patients with autochthonous (n = 131) and travel-associated (n = 142)*Giardia *infections, Germany, 2007-2008

	Autochthonous infections	Travel-associated infections	
	n (%)*	n (%)*	**p-value**

**Male sex**	85 (64.9)	68 (48.2)	**0.007**

**Foreign travel last year**	49 (37.4)	91 (64.1)	**0.001**

Low risk travel destinations only	37 (28.2)	30 (21.1)	0.205

High risk travel destinations	12 (9.16)	61 (43.0)	**0.001**

**Median age (range), years**	41 (0-92)	35 (1-81)	**0.046 **^w^

**Working (age 17-65 years)**	59 (72.8)	83 (78.3)	0.394

**Born in foreign countries**	17 (14.5)	14 (11.7)	0.566

**Residency**			0.571^w^

< 5,000 inhabitants	11 (9.0)	15 (11.0)	

5,000-20,000 inhabitants	17 (13.9)	21 (15.4)	

> 20,000-100,000 inhabitants	29 (23.8)	31 (22.8)	

> 100,000 inhabitants	68 (54.4)	69 (50.7)	

**Clinical manifestations**			

Diarrhoea	109 (83.2)	122 (85.9)	0.615

Bloating	94 (71.8)	103 (72.5)	0.893

Cramps	81 (61.8)	93 (65.5)	0.614

Loss of appetite	67 (51.2)	79 (55.6)	0.469

Nausea	65 (49.6)	69 (48.6)	0.904

Irregular stools	47 (38.2)	72 (54.1)	**0.012**

Fever	40 (32.5)	32 (24.2)	0.165

Vomiting	39 (29.8)	36 (25.3)	0.420

**Hospitalization and workdays missed**			

Hospitalization required	18 (15.1)	5 (3.7)	**0.002**

Sick-leave required	34 (37.0)	38 (35.5)	0.883

**Laboratory Methods**			

Microscopy	51 (38.9)	73 (51.4)	**0.040**

Antigen test (ELISA, IFT)	96 (73.3)	89 (62.7)	0.070

**Reason for laboratory examination**			

Symptoms	124 (94.7)	132 (92.7)	0.623

Contact positive	1 (0.8)	5 (3.5)	0.216

Asylum seeker screening	0 (0)	0 (0)	

Other diseases	4 (3.1)	5 (3.5)	1.000

**Host factors**			

Impaired Immunity	15 (11.8)	2 (1.5)	**0.001**

On antacid therapy	18 (14.0)	10 (7.5)	0.110

Vegetarian	5 (4.2)	5 (3.8)	1.000

**Contact with persons (for adult cases only)**			

Changing diapers for children	15 (13.8)	11 (9.1)	0.301

persons	4 (3.6)	5 (4.1)	1.000

**Children in household**			

Children 0-2 years	20 (15.3)	10 (7.1)	**0.034**

Children 3-6 years	27 (20.6)	23 (16.2)	0.353

Children 7-19 years	40 (30.5)	39 (27.5)	0.595

**Animal contacts**			

Living on a farm	0 (0)	0 (0)	

Owning pets	42 (35.0)	31 (23.0)	**0.038**

*The percentage refers to the proportion among participants who either answered yes or no (i.e. missing and don't-know answers excluded), if not otherwise specified.

^W ^Wilcoxon ranksum test.

The proportion of cases living in communities of over 100,000 inhabitants (54% versus 51%) and the proportion of cases born in foreign countries (15% versus 12%) did not differ significantly between autochthonous and travel-associated cases (Table 1).

Diarrhoea, bloating and abdominal cramps were the most frequently cited clinical symptoms for both travel-associated and autochthonous cases; although autochthonous cases were more likely to be hospitalised (15%, p = 0.002). Among the hospitalised cases, the median age was 38 years (range 3-92); ten (43.5%) were male, four (17.4%) were immunocompromised and five (21.7%) were taking antacid medication.

Autochthonous cases were more likely to report impaired immunity (12% compared to 2% of travel associated cases), to live with children aged less than 3 years (15%) and to own pets (35%) than travel-associated cases.

Of the 142 travel-associated cases, 29 (20%) reported travel to India; ten to Egypt (7%); seven (5%) to Turkey and Thailand respectively, and five (4%) to Nepal, Ecuador and Italy, respectively. Only 6 cases (4%) had travelled to one of the nine countries neighbouring Germany.

### Case-Control study into risk factors for autochthonous symptomatic Giardiasis

In total, 120 cases and 240 matched controls were included in the case-control study.

**Figure 1:**
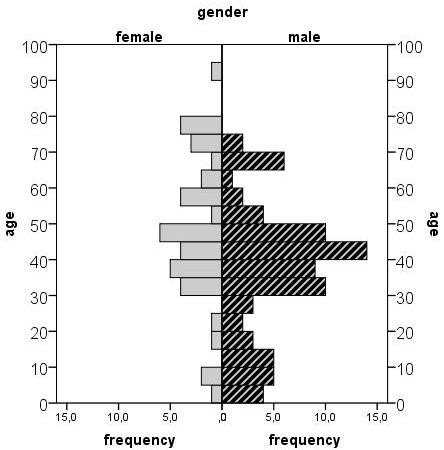
**Age and sex distribution of authochthonous cases (n = 120) included in the case-control study**.

Among the cases, 67% were male (Figure 1), compared to 40% of controls. Because of matching for age groups, 5% of each population were aged 0-5 years, 12.5% were aged 6-19 years and 82.5% were aged 20 years and older.

Travel to foreign countries during the 12 months before the defined incubation period was reported by 45 (38%) of cases compared to 125 (52%) of controls. Contact with someone with diarrhoea was reported by 12 cases (12.9%) compared to 31 controls (16.2%). In univariate analysis, cases were more likely than controls to be male, have an impaired immune system, use antacid drugs and eat green salad daily (Table 2). Persons gardening, hiking, swimming or participating in water sports, drinking tap water daily, travelling abroad in the 12 months before onset of symptoms and having contact with animals appeared to be less likely to fall ill with giardiasis. Migration status, changing children's diapers, caring for elderly or handicapped persons, contact with someone with diarrhoea, living with children and owning pets were all not associated with symptomatic disease. For the 79% of cases and 82% of controls who provided information on the type of occupation, no significant differences in occupation were detected.

**Table 2:** Prevalence and univariate analysis of exposures and potential risk factors among cases with autochthonous *Giardia *infection (n = 120) and matched controls (n = 240), Germany, 2007-2008

	Cases	Controls		
	n (%)*	n (%)*	OR^MH^**	**95%CI**

**Male sex**	80 (66.7)	95 (39.6)	**2.99**	**1.86-4.78**

**Foreign travel last year**	45 (37.5)	125 (52.0)	**0.54**	**0.34-0.86**

Low risk travel destinations only	34 (28.3)	92 (38.3)	0.62	0.38-1.01

High risk travel destinations	11 (9.17)	32 (13.3)	0.66	0.32-1.35

**Working (age 17-65 years)**	57 (60.0)	111 (56.6)	1.73	0.87-3.46

**Contact with persons with diarrhoea**	12 (12.9)	31 (16.1)	0.73	0.34-1.56

**Changing diapers for children**	14 (13.7)	38 (18.1)	0.64	0.32-1.28

**Caring for disabled or elderly persons**	4 (4.0)	14 (6.7)	0.59	0.19-1.81

**Owning pets**	40 (36.3)	92 (39.8)	0.87	0.53-1.41

**Host factors**				

Impaired Immunity	13 (11.2)	7 (3.0)	**5.47**	**1.76-17.0**

On antacid therapy	16 (13.4)	16 (6.8)	**2.18**	**1.04-4.59**

**Household structure**				

children 0-2 years	18 (15.0)	23 (9.6)	1.80	0.88-3.68

children 3-6 years	26 (21.7)	48 (20.0)	1.14	0.62-2.11

children 7-19 years	34 (28.4)	83 (34.6)	0.69	0.40-1.19

**Eating, Drinking**				

Vegetarian food only	5 (4.5)	10 (4.3)	1.25	0.41-3.82

Green salad, daily	26 (21.7)	25 (10.4)	**2.5**	**1.33-4.69**

Raw vegetables, daily	32 (26.7)	75 (31.3)	0.77	0.45-1.31

Raw milk at all	2 (1.7)	10 (4.4)	0.41	0.09-2.03

Mineral water daily	88 (73.3)	170 (70.8)	1.14	0.69-1.88

Tap water daily	36 (30.0)	111 (46.3)	**0.44**	**0.26-0.74**

**Swimming/water sports**	29 (24.4)	77 (34.1)	**0.58**	**0.34-1.00**

**Contact with waste water**	3 (2.6)	9 (4.0)	0.62	0.15-2.53

**Outdoor activities**	41 (36.6)	151 (69.6)	**0.12**	**0.06-0.26**

Fishing	1 (0.9)	9 (4.1)	0.18	0.02-1.50

Gardening	24 (21.4)	87 (40.1)	**0.27**	**0.14-0.54**

Farming	0	6 (2.8)		

Hunting	0	3 (1.4)		

Hiking	17 (15.2)	64 (29.5)	**0.37**	**0.19-0.72**

Camping	8 (7.1)	8 (3.7)	1.64	0.57-4.75

**Living on a farm**	0	8 (3.4)		

**Contact with animals**	61 (51.3)	147 (62.0)	**0.60**	**0.37-0.98**

Cat	31 (26.0)	77 (32.4)	0.72	0.44-1.19

Dog	41 (34.5)	81 (34.1)	1.02	0.61-1.70

Bird	10 (8.4)	16 (6.8)	1.29	0.52-3.24

Guinea pig	1 (0.8)	6 (2.5)	0.33	0.04-2.77

Rabbit/hare	8 (6.7)	17 (7.2)	0.93	0.38-2.28

Horse	4 (3.4)	16 (6.8)	0.47	0.15-1.46

Cow	2 (1.7)	14 (5.9)	0.25	0.05-1.16

Pig	0	4 (1.7)		

Sheep	2 (1.7)	2 (0.8)	2.00	0.28-14.2

Goat	1 (0.8)	1 (0.4)	2.00	0.13-31.9

*The percentage refers to the proportion among participants who either answered yes or no (i.e. missing and don't-know answers excluded).

**Mantel Haenszel odds ratio

In conditional logistic regression, male sex, having impaired immunity and eating green salad daily remained independently associated with autochthonous symptomatic giardiasis. Those who gardened were less likely to develop disease (Table 3). Interaction variables constructed as the product of any two of these factors showed no significant influence.

**Table 3:** Final multivariate model (conditional logistic regression) for autochthonous *Giardia *infections, Germany, 2007-2008, N = 299

	Cases	Controls			
	n (%)*	n (%)*	**p-value**	aOR***	**95%CI**

**Male sex**	71 (66.4)	78 (40.6)	0.001	**2.5**	**1.4 - 4.4**

**Host factors**					

Impaired Immunity	12 (11.2)	4 (2.1)	0.012	**15.3**	**1.8 - 127.0**

**Eating**					

Green salad, daily	24 (22.4)	17 (8.9)	0.017	**2.9**	**1.2 - 7.2**

**Outdoor activities**					

Gardening	24 (22.4)	80 (41.7)	< 0.001	**0.26**	**0.12 - 0.55**

*The percentage refers to the proportion among participants who either answered yes or no (i.e. missing and don't-know answers excluded).

***adjusted odds ratio

## Discussion

The results of our study demonstrate that giardiasis in Germany is frequently acquired indigenously, with only about 50% of cases reporting travel to foreign countries during the three weeks before onset of symptoms. This is consistent with the data reported to the German surveillance system on the country of infection [8]. The notification rates for giardiasis in European countries vary greatly [36] and people returning from travel abroad probably import a significant proportion of infections in some countries [37].

Chronic infection and asymptomatic carriage from previous travel could lead to underreporting of travel-associated cases. However, in our study more controls than cases had travelled to foreign countries in the year before the defined incubation period.

In regression analysis, we found three independent risk factors for giardiasis in Germany. Among these, the risk associated with the consumption of green salad has previously been documented in the literature [25-29]. The food industry is becoming increasingly aware of the potential for contamination of foodstuffs with *Giardia lamblia *[38,39]. Green salad and similar fresh produce is consumed with minimal preparation, making it a potential vehicle of infection. *Giardia *cysts have previously been isolated from salad [26,38,40-42]. Contamination of salads may be due to the use of faecal contaminated irrigation water [43] or to the preparation of the salad by infected food handlers [25,41,44]. Contamination of salad by infected animals could be another plausible route of transmission.

Giardiasis has previously been reported among the immunocompromised [7,45-47] and in our study impaired immunity was also associated with infection. The causes of impaired immunity among our cases were pancreatic cancer, stem cell therapy, intestinal disease/surgery, HIV, Morbus Crohn, diabetes, cancer and asthma. It is not clear whether these individuals are more susceptible to the infection or whether they are more likely to develop symptomatic disease. Furthermore, their underlying condition may increase their likelihood of submitting a specimen and being laboratory-confirmed as a *Giardia *case. It should be noted that although the immunocompromised had a high OR for *Giardia *infection, they still only accounted for a small proportion of cases.

Male sex was also associated with symptomatic giardiasis in our study. This sex-related difference may be attributable in part to sexual contact among men who have sex with men (MSM). However, our data as well as the complete surveillance data for Germany in 2007 show that males in nearly every age group were more affected than females, except for the 15-24 year olds, where the incidence among women was slightly higher [8]. Similar data have been reported from other countries in the European Union [36] and from the United States [20], which suggests the influence of other factors such as differences in hygiene (e.g. washing hands).

Contact with fresh water or drinking of tap water were not associated with disease in our study. In Germany, the tap-water supply comes from a variety of sources and is distributed through many different waterworks. Private wells have become very rare. Deficiencies in the reduction of *Giardia *cysts in smaller sewage treatment plants in Germany have been documented [48]. As our study covered only a sample of all the districts in Germany, we were not able to detect risks associated with different local water supplies. Therefore we cannot definitively exclude water as a risk factor for infection.

Likewise we found no association between animal contacts and disease. This is in accordance with recent reports where the genotypes of *Giardia *spp. isolated from animals did not cause symptoms in humans [49].

Travel outside Germany during the exposure period was reported by about half our cases. The Indian subcontinent was cited as the main destination in our enhanced surveillance in accordance with the findings of an earlier description of German travel-associated *Giardia *cases [50]. As travel to Asia accounted for only 6.6% of all foreign travel by Germans during 2008 [51], this indicates an increased risk of acquiring symptomatic giardiasis with travel to India.

The finding that autochthonous cases were hospitalized more often than the travel-associated cases could possibly be explained by the older age and partially reduced immune response of this group.

Some limitations of this study should be considered. Although we aimed to recruit controls representative of the source population of cases, we observed that a high proportion of young people and those living in large cities did not have a publicly registered telephone number. Furthermore, females, the elderly and those living in rural areas were more likely to participate. We therefore had a higher proportion of female controls. This selection bias emphasized the sex differences between cases and controls and could explain why some exposures such as gardening, hiking, animal contacts and living in rural areas were more frequent among the control group.

In our case control study we looked for risk factors for clinical giardiasis. As we have no information on asymptomatic carriage of *Giardia *trophozoites in cases before our defined exposure period, it is possible that in some cases we looked for risk factors for the expression of symptoms following asymptomatic carriage rather than for risk factors related to the transmission of the pathogen. It is therefore possible that some cases became infected with *Giardia *long before our defined exposure period.

In our controls, too, we had no information on asymptomatic carriage. This might have led to misclassification and some of the controls might have met our case definition, if they had been laboratory tested.

Unfortunately we were not able to include enough cases for risk factor analyses in subgroups such as children or MSM.

## Conclusions

In Germany, a substantial proportion of *Giardia lamblia *cases is due to autochthonous infections. Persons with immune deficiencies appear to be more likely to have symptomatic giardiasis. Physicians should consider *Giardia *infections not only in travel-associated cases, but also in those with no history of travel abroad, especially in patients with immune deficiencies.

Our study provides evidence that contaminated green salad may be an important vehicle for those infections. Information campaigns on this food-borne risk should particularly focus on persons with immune deficiencies.

In our study, contacts with animals and surface water were not identified as risk factors for autochthonous sporadic human giardiasis. Further studies are needed to clarify the potential for zoonotic transmission and the role of surface water.

Outbreak investigations especially among children and male adults should be conducted to learn more about risk factors for these groups in Germany.

## Competing interests

The authors declare that they have no competing interests.

## Authors' contributions

WE, KA and KS designed the study. WE organized the data-collection. MAH and WE performed the data analysis. WE drafted the manuscript. All authors discussed the results and revised the manuscript. All authors read and approved the final manuscript.

## Pre-publication history

The pre-publication history for this paper can be accessed here:

http://www.biomedcentral.com/1471-2458/10/41/prepub
